# Quality of life after breast (reconstructive) surgery—an interim analysis of a prospective three-arm clinical trial with a 10-year follow-up (REKO 001)

**DOI:** 10.1007/s00404-026-08387-2

**Published:** 2026-04-07

**Authors:** B. Boeer, J. Kandzi, B. Schoenfisch, M. Marx, S. Guergan, I. Gruber, C. Roehm, G. Helms, A. Hartkopf, S. Y. Brucker, M. Hahn

**Affiliations:** 1 Department of Women’s Health, University Hospital of Tuebingen, Calwerstraße 7, 72076 Tuebingen, Germany; 2 Clinic for Plastic Surgery, Elblandklinikum Radebeul, Radebeul, Germany

**Keywords:** Breast cancer, Breast surgery, Reconstructive surgery, Quality of life

## Abstract

**Background:**

Long-term health-related quality of life (HRQoL) plays an increasingly important role in breast cancer treatment. Women who are not eligible for breast-conserving therapy frequently opt for reconstruction, but data on long-term health-related quality of life comparing different reconstructive surgeries is rare.

**Methods:**

REKO 001-trial is a single-center, three-arm study with a prospective longitudinal design and 10-year follow-up, investigating which surgery is associated with the highest quality of life. Between January 2017 and March 2022, 227 patients underwent either mastectomy (74), implant-based (77), or Deep Inferior Epigastric Perforator (DIEP) or Fascio-Cutaneous Infragluteal (FCI) free flap (76) reconstruction.

This initial follow-up analysis compared HRQoL (BREAST-Q, FACT-B) and postoperative complications 5 months after surgery.

**Results:**

The DIEP/FCI group had the lowest preoperative BREAST-Q scores, but was the only group to show significant increases in many HRQoL domains within the first 5 months postoperatively. The mastectomy group showed significant decreases in some HRQoL domains. The implant-based group started with the highest BREAST-Q scores in most domains and had higher HRQoL scores than the mastectomy group. Radiotherapy and higher BMI significantly reduced HRQoL in some domains.

The number of complications requiring inpatient treatment or reoperation within the first 5 months increased with extent of surgery (p = 0.037).

**Conclusion:**

For patients opting for DIEP/FCI reconstruction, an increase in HRQoL can be seen within the first 5 months after surgery, but they must accept the higher risk of complications and a decrease in abdominal well-being. Further follow-ups after 2, 5, 7, and 10 years are underway.

## What does this study add to the clinical work?

**Table:** 

This initial analysis of the REKO 001 trial demonstrates that patients undergoing autologous reconstruction (DIEP/FCI) experience an improvement in their quality of life following a period of just five months, despite encountering a higher complication rate in comparison to patients who have undergone mastectomy or heterologous reconstruction. Consequently, this paper offers significant insights for individual surgical decision-making and preoperative consultations in breast cancer treatment.	

## Introduction

Survival rates in early breast cancer are relatively good nowadays compared with other malignancies [1]. Long-term survivors consistently emphasize the need for robust research on health-related quality of life (HRQoL) [2]. HRQoL is an important prognostic factor and health-related satisfaction is significantly associated with survival in breast cancer patients, regardless of disease stage [3]. One in eight women can be expected to be diagnosed with breast cancer during their lifetime. Consequently, the epidemiological relevance of this disease and the assessment of applied therapies is correspondingly high. Approximately 30% of women who are not eligible for breast-conserving surgery choose reconstructive procedures. In addition, reconstructive surgery is performed in carriers of germline pathogenic variants after risk-reducing mastectomy [4]. The Oncoplastic Breast Consortium addressed major gaps in data on HRQoL in oncoplastic breast surgery [5]. To date, no longitudinal study has compared HRQoL following mastectomy, implant-based reconstruction, and autologous reconstruction over a 10-year period.

The REKO 001-trial investigates HRQoL with a long-term follow-up of 10 years comparing mastectomy versus implant-based versus autologous reconstruction (Deep Inferior Epigastric Perforator (DIEP) or Fascio-Cutaneous Infragluteal (FCI) free flap). The main objective of the REKO 001-trial is to evaluate the quality of life in the long term depending on the type of surgery. The primary objective of this interim analysis was to assess HRQoL 5 months after surgery. The secondary aim was to record the number of follow-up operations and inpatient readmissions within 5 months after surgery.

## Methods

### Study design

The REKO 001-trial is a single-center, three-arm, non-randomized trial conducted at the Department of Women’s Health Tuebingen with a prospective longitudinal design. All patients scheduled for breast surgery due to breast cancer or carriers of germline pathogenic variants were screened and enrolled prior to surgery. Patients were allocated to one of three groups according to the planned procedure: mastectomy, implant-based reconstruction, or DIEP/FCI reconstruction. Patient recruitment started in January 2017 and lasted until March 2022 with follow-up over 10 years. Female patients between the ages of 18 and 70 are asked to complete HRQoL questionnaires six times during this period: the day before surgery (T1), 5 months (T2), 2 years, 5 years, 7.5 years, and 10 years after surgery. Exclusion criteria were lack of patient consent and a life expectancy less than 10 years.

The sample size was calculated based on a moderate effect size of d = 0.5 for quality of life. With a power of 0.8 and a significance level of 0.05, the required sample size was found to be n = 64 per study arm. Due to the 10-year follow-up period of the REKO 001-trial, a 20% loss to follow-up is expected. Therefore, the total sample size was set at 230 patients (3 × 64 × 120%).

The study was approved by the local ethics committee (University of Tuebingen, 790/2016BO2) and registered at the German Clinical Trials Register (DRKS00014803).

### Questionnaires

HRQoL was measured by the internationally established BREAST-Q and FACT-B questionnaires [6]. The endpoints assessed by the BREAST-Q questionnaire are psychosocial, sexual, and physical outcomes in addition to satisfaction with the surgical outcome of breast surgery, general outcome, and the treatment process [7]. The Functional Assessment of Cancer Therapy-Breast (FACT-B) questionnaire assesses therapy-specific non-surgical effects on HRQoL in breast cancer patients. FACT-B is derived from the “Functional Assessment of Cancer Therapy-General” Questionnaire (FACT-G) and becomes FACT-B by adding a breast cancer-specific module with ten items (Breast Cancer Subscale) [8].

### Statistical methods

Data were documented in a REDCap database (Research Electronic Data Capture). Analysis was performed with the aid of IBM® SPSS® Statistics Version 28 software. Both questionnaires were evaluated according to the manuals, with BREAST-Q using QScore software (version 1). The analysis of variance (ANOVA) was used to compare continuous variables such as age, BMI, and questionnaire scores between groups at T1 and T2. A Bonferroni post hoc analysis was performed on significant results. The results of the questionnaires between time points T1 and T2 within the groups were analyzed using the paired *t *test. Unpaired *t *tests were performed for comparisons of only two groups (e.g., Satisfaction with Outcome). The Chi^2^ test was used for nominal variables. The influence of various factors (age, body mass index etc.) on the FACT-B and BREAST-Q scores was estimated using multiple regression models (Program R, version 4.2.2 with the packages nlme and r2glmm). The coefficient of determination R^2^ was specified for the models at time T1. To evaluate the data over time (T1 and T2), mixed multiple regression models were formulated with the patient as a random factor. Here, pseudo R^2^ values were calculated for the model quality using the r2beta() function.

## Results

**Fig. 1:**
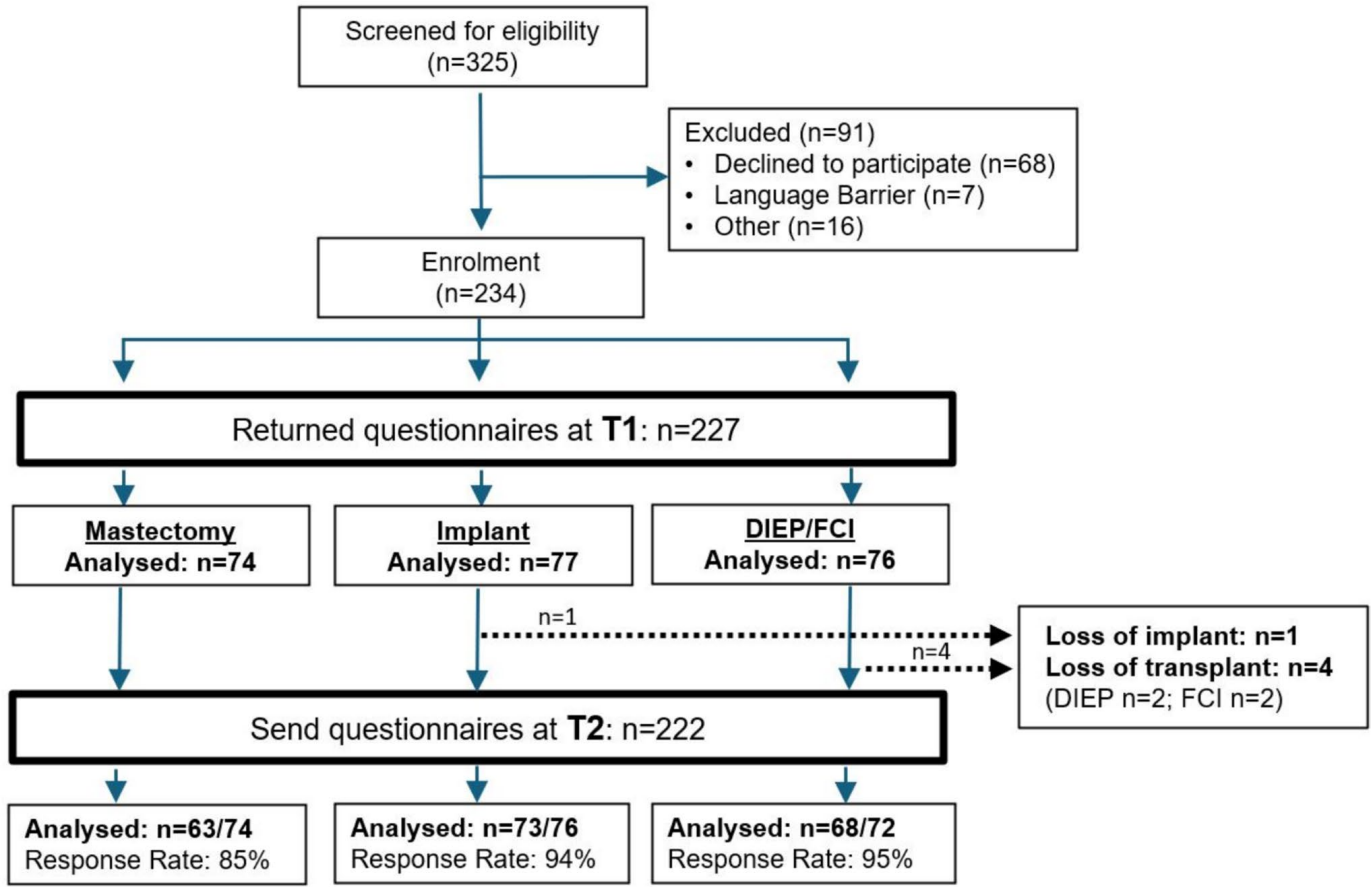
Diagram of patient enrolment

A total of 325 patients were screened. After exclusion of 91 patients, informed consent was obtained from 234 patients. Of the 234 patients enrolled in the study, 227 returned the initial questionnaire at T1, while 7 were lost to follow-up. Prior to T2, five patients were excluded because of implant or transplant loss. A total of 204 questionnaires were returned at T2; however, 23 were lost to follow-up between T1 and T2, resulting in an overall loss of 30 between enrolment and T2 (Fig. 1).

Age was similar for the DIEP/FCI and implant group (*p* = 0.196), but both groups were significantly younger than the mastectomy group (*p* < 0.001 and *p* = 0.008, respectively) (Table 1). For carriers of germline pathogenic variants, age was significantly lower in the implant than in the DIEP/FCI group (35.4 vs. 46.3 years, *p* = 0.002). Body mass index (BMI) values were not significantly different between the groups (*p* = 0.118). There was a significant difference in the proportion of active smokers (*p* = 0.042) and regarding previous breast surgeries (*p* < 0.001) between groups (Table 1). 98.7% in the DIEP/FCI group had undergone a surgical intervention in the past compared to 42.9% in the implant and 25.7% in the mastectomy group (*p* < 0.001). Overall, 15% (33/227) of patients carried a breast cancer-associated germline mutation. Among these patients, 12% (4/33) underwent bilateral prophylactic surgery within the same procedure (two implant-based, two DIEP/FCI). Twenty-nine of the thirty-three carriers (88%) had a diagnosis of breast cancer. Among them, 17 (52%) underwent reconstruction, and 12 (36%) had either a second surgery or prophylactic contralateral surgery. The prevalence of germline pathogenic variants differed significantly between groups, with the highest proportions in the implant and DIEP/FCI cohorts (*p* < 0.001). The frequency of all additional therapies that were applied or completed prior to study inclusion differed significantly between the groups (all *p* < 0.001). Patients in the DIEP/FCI group had mostly completed radiotherapy (65.8%) and chemotherapy (65.8%) and started endocrine therapy (72.4%) before surgery. During the 5-month follow-up, 47.3% of the mastectomy group and 11.7% of the implant group had radiotherapy, whereas none of the patients in the DIEP/FCI group were treated with radiotherapy (*p* < 0.001). Adjuvant chemotherapy was given to 35.1% of the mastectomy group and 19.5% of the implant group, compared with only one patient in the DIEP/FCI cohort (1.3%) (*p* < 0.001).

**Table 1:** Patient characteristics

Patient characteristics *n* = 227	Mastectomy group *n* = 74	Implant group *n* = 77	DIEP/FCI group *n* = 76 (*n* = 64 DIEP; *n* = 12 FCI)	*p* value
	Number (percentage) respectively mean (SD)	Number (percentage) respectively mean (SD)	Number (percentage) respectively mean (SD)	
Age [years]	52.3 (11.2)	44.9 (9.4)	49.5 (7.2)	< 0.001‡
BMI [kg/m^2^]	26.2 (7.0)	24.5 (4.7)	25.8 (4.3)	0.118‡
Smoker at time of surgery	6 (8.1%)	6 (7.7%)	0 (0.0%)	0.042*
Previous surgery	19 (25.7%)	33 (42.9%)	75 (98.7%)	< 0.001*
Mastectomy	0 (0.0%)	0 (0.0%)	42 (55.3%)	
Mastectomy + expander	0 (0.0%)	3 (3.9%)	0 (0.0%)	
SSM/NSM + implant	0 (0.0%)	4 (5.2%)	27 (35.6%)	
BCT	11 (14.8%)	9 (11.7%)	1 (1.3%)	
Breast augmentation	0 (0.0%)	4 (5.2%)	2 (2.6%)	
SLNB	3 (4.1%)	13 (16.9%)	0 (0.0%)	
Reduction mammoplasty Other	0 (0.0%) 5 (6.8%)	0 (0.0%) 0 (0.0%)	1 (1.3%) 2 (2.6%)	
Mutation carrier	1 (1.4%)	20 (25.6%)	12 (15.8%)	< 0.001*
Major complications	3 (4.1%)	9 (11.7%)	13 (17.1%)	0.037*
Infection	0 (0.0%)	0 (0.0%)	2 (2.6%)	
Seroma	0 (0.0%)	0 (0.0%)	2 (2.6%)	
Hematoma	1 (1.4%)	7 (9.1%)	3 (4.0%)	
Implant/transplant loss	0 (0.0%)	1 (1.3%)	4 (5.3%)	
Wound dehiscence	2 (2.7%)	1 (1.3%)	2 (2.6%)	
Chemotherapy	*n* = 48	*n* = 39	*n* = 51	
Before T1	29 (39.2%)	24 (31,2%)	50 (65.8%)	< 0.001*
Between T1–T2	26 (35.1%)	15 (19.5%)	1 (1.3%)	< 0.001*
Radiation therapy	*n* = 43	*n* = 13	*n* = 50	
Before T1	8 (10.8%)	4 (5.2%)	50 (65.8%)	< 0.001*
Between T1–T2	35 (47.3%)	9 (11.7%)	0 (0.0%)	< 0.001*
Endocrine therapy	*n* = 46	*n* = 46	*n* = 55	
Before T1	9 (12.2%)	8 (10.4%)	55 (72.4%)	< 0.001*
Between T1-T2	40 (54.1%)	42 (54.5%)	49 (64.5%)	0.342

*SSM* skin-sparing mastectomy, *NSM* nipple-sparing mastectomy, *BCT* breast-conserving therapy, *SLNB* sentinel lymph node biopsy

‡ANOVA

*Chi^2^ test

### Complications

Overall, 11.0% (25/227) of patients had complications such as hematoma or implant–transplant loss within 5 months after the procedure that required an inpatient stay or reoperation (Table 1). The highest rate of complications was in the DIEP/FCI group (17.1%) followed by the implant-based group (11.7%) and mastectomy group (4.1%, *p* = 0.037). Complete loss of the reconstruction occurred in 1 of 77 cases (1.3%) in the implant-based group and in 4 of 76 cases (5.3%) in the DIEP/FCI group. In the long-term follow-up of the REKO 001-trial, these cases will be observed and analyzed separately as a conversion group.

### Breast-Q

**Fig. 2:**
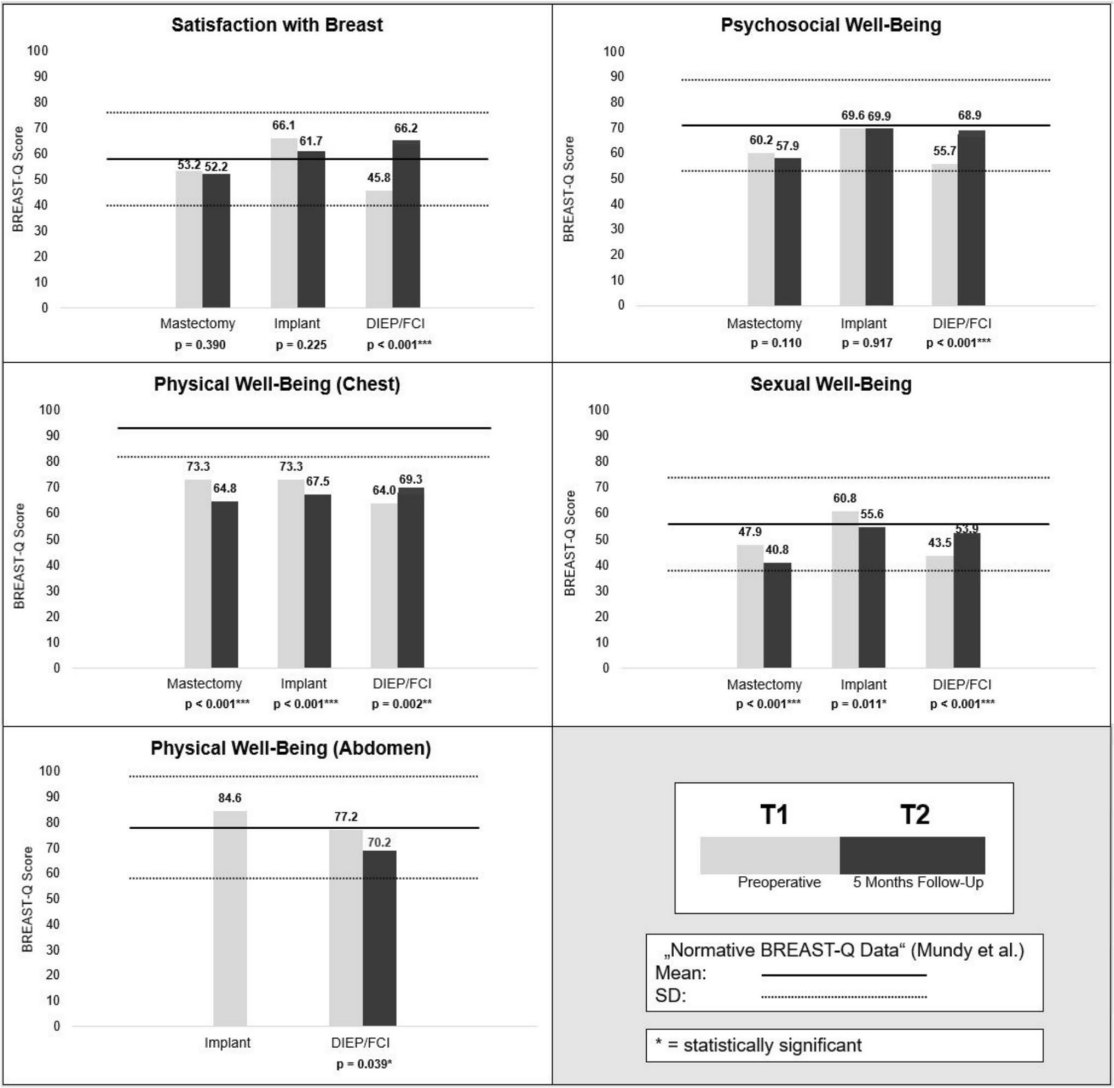
Overview of BREAST-Q results and changes within groups over time

Preoperatively at T1, patients in the implant group had the highest BREAST-Q scores, while scores were lowest in the DIEP/FCI group in all domains (Table 2; Fig. 2). The difference in scores was significant in most domains. Four domains of the BREAST-Q are included in the preoperative (T1) as well as in the 5-month postoperative (T2) questionnaire. In the DIEP/FCI group, all four domains improved from T1 to T2. In contrast, scores declined in the mastectomy and implant groups, except for a minimal increase in Psychosocial Well-Being in the implant group. The domain Physical Well-Being (Abdomen) was only part of the reconstructive questionnaire. The implant-based group had higher scores at T1; scores in the DIEP/FCI group dropped significantly from T1 to T2. At T2, the mastectomy group showed significantly lower scores than the other groups in most domains, while the scores of the implant and DIEP/FCI groups were not significantly different (Fig. 2).

**Table 2:** Overview of BREAST-Q results and differences between groups

Breast-Q domain	Group	Mean	SD	*p* value (ANOVA with post hoc analysis)	
*T1*					
Satisfaction with Breast	Mastectomy	53.2	21.0	Mastectomy vs. DIEP/FCI	0.155
	Implant	66.1	24.6	Mastectomy vs. Implant	0.001
	DIEP/FCI	45.8	19.9	Implant vs. DIEP/FCI	< 0.001
Psychosocial Well-Being	Mastectomy	60.2	16.9	Mastectomy vs. DIEP/FCI	0.419
	Implant	69.6	20.9	Mastectomy vs. Implant	0.006
	DIEP/FCI	55.7	17.7	Implant vs. DIEP/FCI	< 0.001
Physical Well-Being (Chest)	Mastectomy	73.3	13.7	Mastectomy vs. DIEP/FCI	< 0.001
	Implant	73.3	14.5	Mastectomy vs. implant	1.000
	DIEP/FCI	64.0	15.7	Implant vs. DIEP/FCI	< 0.001
Physical Well-Being (Abdomen)	Mastectomy	N/A			
	Implant	84.6	15.9	Implant vs. DIEP/FCI	0.014^b^
	DIEP/FCI	77.2	18.8		
Sexual Well-Being	Mastectomy	47.9	20.2	Mastectomy vs. DIEP/FCI	0.577
	Implant	60.8	18.2	Mastectomy vs. implant	< 0.001
	DIEP/FCI	43.5	19.8	Implant vs. DIEP/FCI	< 0.001
*T2*					
Satisfaction with Breast	Mastectomy	52.2	16.6	Mastectomy vs. DIEP/FCI	< 0.001
	Implant	61.7	16.8	Mastectomy vs. implant	0.005
	DIEP/FCI	66.2	17.8	Implant vs. DIEP/FCI	0.379
Satisfaction with Outcome	Mastectomy	N/A		Implant vs. DIEP/FCI	
	Implant	76.3	19.0		0.521^b^
	DIEP/FCI	76.5	19.8		
Psychosocial Well-Being	Mastectomy	57.9	15.5	Mastectomy vs. DIEP/FCI	0.001
	Implant	69.9	19.3	Mastectomy vs. implant	< 0.001
	DIEP/FCI	68.9	17.8	Implant vs. DIEP/FCI	1.000
Physical Well-Being (Chest)	Mastectomy	64.8	14.2	0.161^a^	
	Implant	67.5	13.9		
	DIEP/FCI	69.3	12.9		
Sexual Well-Being	Mastectomy	40.8	18.7	Mastectomy vs. DIEP/FCI	0.001
	Implant	55.6	20.5	Mastectomy vs. implant	< 0.001
	DIEP/FCI	53.9	19.3	Implant vs. DIEP/FCI	1.000

^a^ANOVA without significance, no post hoc analysis

^b^Unpaired *t* test

### FACT-B

**Fig. 3:**
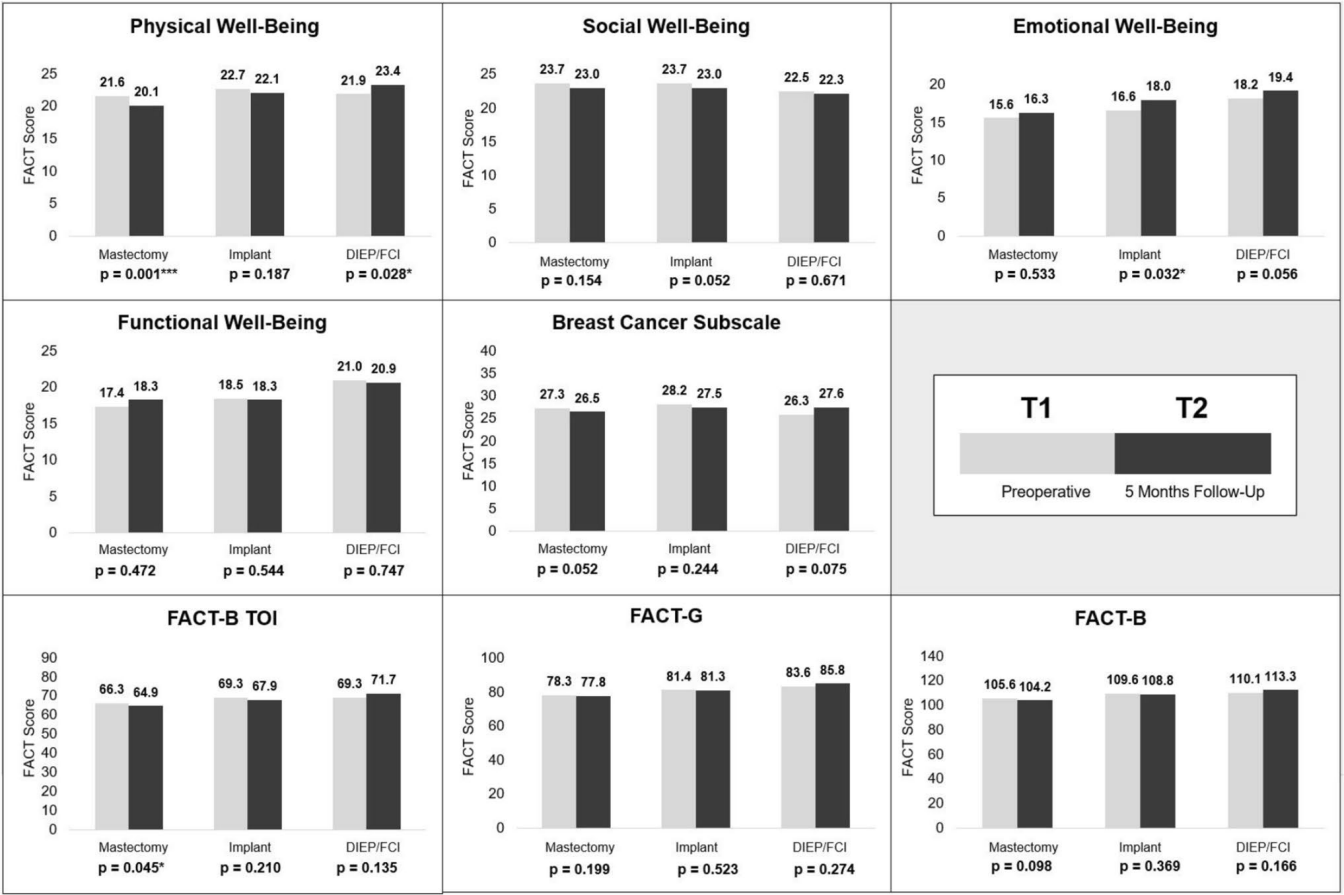
Overview of FACT-B results and changes within groups over time

Preoperatively, FACT-B scores were quite similar in all groups; only the domains Emotional and Functional Well-Being were significantly different in the mastectomy and DIEP/FCI group, with higher scores in the latter. From T1 to T2, all sum scores showed an increase in the DIEP/FCI group, whereas they declined in both the mastectomy and implant group. However, these effects were not statistically significant. All groups showed an increase in Emotional Well-Being from T1 to T2 (Fig. 3). At T2, the mastectomy group had the lowest results in all sum scores, whereas the DIEP/FCI group had the highest. The differences in the FACT-B total sum scores 5 months after surgery were not significant between the groups (Table 3; Fig. 3).

**Table 3:** Overview of FACT-B results and differences between groups

FACT-B domain	Group	Mean	SD	*p* value (ANOVA with post hoc analysis)			
*T1*							
Physical Well-Being	Mastectomy	21.6	5.2	0.419^a^			
	Implant	22.7	5.4				
	DIEP/FCI	21.9	5.2				
Social Well-Being	Mastectomy	23.7	4.4	0.173^a^			
	Implant	23.7	4.8				
	DIEP/FCI	22.5	4.6				
Emotional Well-Being	Mastectomy	15.6	5.2	Mastectomy vs. DIEP/FCI		0.004	
	Implant	16.6	5.2	Mastectomy vs. implant		0.705	
	DIEP/FCI	18.2	4.0	Implant vs. DIEP/FCI		0.105	
Functional Well-Being	Mastectomy	17.4	6.1	Mastectomy vs. DIEP/FCI		< 0.001	
	Implant	18.5	6.1	Mastectomy vs. implant		0.772	
	DIEP/FCI	21.0	5.0	Implant vs. DIEP/FCI		0.024	
Breast cancer subscale	Mastectomy	27.3	6.9	0.238^a^			
	Implant	28.2	7.0				
	DIEP/FCI	26.3	6.6				
FACT-B TOI	Mastectomy	66.3	15.7	0.376^a^			
	Implant	69.3	16.1				
	DIEP/FCI	69.3	14.0				
FACT-G	Mastectomy	78.3	15.9	0.120^a^			
	Implant	81.4	16.8				
	DIEP/FCI	83.6	14.1				
FACT-B	Mastectomy	105.6	20.9	0.348^a^			
	Implant	109.6	21.9				
	DIEP/FCI	110.1	19.0				
T2							
Physical Well-Being	Mastectomy	20.1	5.6	Mastectomy vs. DIEP/FCI		0.001	
	Implant	22.1	5.4	Mastectomy vs. implant		0.068	
	DIEP/FCI	23.4	4.1	Implant vs. DIEP/FCI		0.416	
Social Well-Being	Mastectomy	23.0	4.6	0.519^a^			
	Implant	23.0	4.7				
	DIEP/FCI	22.3	5.0				
Emotional Well-Being	Mastectomy	16.3	3.9	Mastectomy vs. DIEP/FCI		< 0.001	
	Implant	18.0	4.9	Mastectomy vs. implant		0.076	
	DIEP/FCI	19.4	3.8	Implant vs. DIEP/FCI		0.174	
Functional Well-Being	Mastectomy	18.3	5.4	Mastectomy vs. DIEP/FCI		0.043	
	Implant	18.3	6.7	Mastectomy vs. implant		1.000	
	DIEP/FCI	20.9	5.4	Implant vs. DIEP/FCI		0.028	
Breast cancer subscale	Mastectomy	26.5	6.4	0.541^a^			
	Implant	27.5	7.3				
	DIEP/FCI	27.6	6.0				
FACT-B TOI	Mastectomy	64.9	15.3	Mastectomy vs. DIEP/FCI		0.040	
	Implant	67.9	17.4	Mastectomy vs. implant		0.788	
	DIEP/FCI	71.7	13.5	Implant vs. DIEP/FCI		0.454	
FACT-G	Mastectomy	77.8	16.1	Mastectomy vs. DIEP/FCI		0.019	
	Implant	81.3	18.3	Mastectomy vs. implant		0.649	
	DIEP/FCI	85.8	14.4	Implant vs. DIEP/FCI		0.337	
FACT-B	Mastectomy	104.2	21.0	0.063^a^			
	Implant	108.8	24.4				
	DIEP/FCI	113.3	18.9				

^a^ANOVA without significance, no post hoc analysis

### Regression analysis

At T1, higher BMI was associated with a significantly lower HRQoL, except for the BREAST-Q domain Physical Well-Being (Chest) (*p* = 0.113). Age had no significant influence on HRQoL in these models (Table 4). Previous radiotherapy appeared to have a significant negative impact on HRQoL in the domain Physical Well-Being (Chest) (*p* < 0.001) and the FACT-B (*p* = 0.006) at T1. Prior chemotherapy was associated with reduced scores in the Sexual Well-Being domain (*p* = 0.044). Smoking status, endocrine therapy, previous breast surgery, and tumor stage had no significant influence on HRQoL in these models (Table 4).

**Table 4:** Regression models

	FACT-B		Breast-Q satisfaction with breast		Breast-Q psychosocial well-being		Breast-Q physical well-being (chest)		Breast-Q sexual well-being	
Variable	Estimate	*p* value	Estimate	*p* value	Estimate	*p* value	Estimate	*p* value	Estimate	*p* value
Intercept	118.46	< 0.001*	76.32	< 0.001*	75.12	< 0.001 *	87.53	< 0.001*	56.20	< 0.001*
Mastectomy	Reference									
DIEP/FCI	7.14	0.118	0.31	0.937	5.09	0.190	− 8.63	0.012*	14.99	< 0.001*
Implant	3.33	0.361	7.31	0.023*	9.85	0.002 *	− 4.78	0.083	20.11	< 0.001*
Age [years]	0.07	0.636	− 0.10	0.403	− 0.09	0.463	− 0.07	0.488	− 0.04	0.763
BMI [kg/m^2^]	-0.52	0.028*	− 0.65	0.002**	− 0.51	0.012 *	− 0.35	0.047*	− 0.62	0.003*
Non-smoker	Reference									
Smoker	1.19	0.841	1.88	0.715	− 2.24	0.655	− 0.61	0.891	− 6.78	0.233
No previous surgery	Reference									
Previous surgery	-4.72	0.163	− 2.69	0.366	− 3.44	0.235	− 1.54	0.549	− 7.53	0.029*
No chemotherapy	Reference									
Chemotherapy	2.76	0.429	1.63	0.594	5.75	0.054	1.76	0.499	4.67	0.167
No radiotherapy	Reference									
Radiotherapy	− 7.07	0.052	− 6.48	0.042*	− 2.77	0.371	0.64	0.815	− 6.78	0.055
No endocrine therapy	Reference									
Endocrine therapy	0.48	0.866	0.48	0.846	0.54	0.823	− 0.11	0.959	7.37	0.006*
Time	-0.76	0.597	4.04	0.026*	2.85	0.030*	− 13.16	< 0.001*	10.78	< 0.001*
Pseudo R^2^	**0.05**		**0.10**		**0.11**		**0.19**		**0.20**	

The significance of bold values is to distinguish it from the other entries

*Statistically significant

The mixed model analysis (Table 5) showed scores significantly increasing with time in two domains of the BREAST-Q regarding Satisfaction with Breast (*p* = 0.019) and Psychosocial Well-Being (*p* = 0.019). Scores in the Physical Well-Being (Chest) domain decreased significantly (*p* = 0.001). In the FACT-B and in most of the domains of the BREAST-Q, quality of life decreased significantly with increasing BMI. The implant group had significantly higher scores compared to the mastectomy group in the domains Satisfaction with Breast (*p* = 0.004), Psychological Well-Being (*p* = 0.011), and Sexual Well-Being (*p* = 0.010). In the DIEP/FCI group, the values compared to the mastectomy group were significantly higher in the FACT-B (*p* = 0.014). Radiotherapy was associated with significantly lower FACT-B scores (*p* < 0.001) and lower scores in the BREAST-Q domains Psychosocial Well-Being (*p* < 0.001), Physical Well-Being (Chest) (*p* = 0.026), and Sexual Well-Being (*p* < 0.001). Higher tumor stage was associated with significantly higher FACT-B scores (*p* = 0.032). Age, smoking status, endocrine therapy, previous breast surgery, and prior chemotherapy showed no significant associations with HRQoL in these models.

**Table 5:** Mixed model analysis

	FACT-B		Breast-Q satisfaction with breast		Breast-Q psychosocial well-being		Breast-Q physical well-being (Chest)		Breast-Q sexual well-being	
Variable	Estimate	*p* value	Estimate	*p* value	Estimate	*p* value	Estimate	*p* value	Estimate	*p* value
Intercept	118.46	< 0.001 *	76.32	< 0.001*	75.12	< 0.001*	87.53	< 0.001*	56.20	< 0.001*
Mastectomy	Reference									
Implant	3.33	0.361	7.31	0.023*	9.85	0.002*	− 4.78	0.083	20.11	< 0.001*
DIEP/FCI	7.14	0.118	0.31	0.937	5.09	0.190	− 8.63	0.012*	14.99	< 0.001*
Age [years]	0.07	0.636	− 0.10	0.403	− 0.09	0.463	− 0.07	0.488	− 0.04	0.763
BMI [kg/m^2^]	− 0.52	0.028 *	-0.65	0.002*	− 0.51	0.012*	− 0.35	0.047*	− 0.62	0.003*
Non-smoker	Reference									
Smoker	1.19	0.841	1.88	0.715	− 2.24	0.655	− 0.61	0.891	− 6.78	0.233
No previous surgery	Reference									
Previous surgery	− 4.72	0.163	− 2.69	0.366	− 3.44	0.235	− 1.54	0.549	− 7.53	0.029*
No chemotherapy	Reference									
Chemotherapy	2.76	0.429	1.63	0.594	5.75	0.054	1.76	0.499	4.67	0.167
No radiotherapy	Reference									
Radiotherapy	− 7.07	0.052	− 6.48	0.042*	− 2.77	0.371	0.64	0.815	− 6.78	0.055
No endocrine therapy	Reference									
Endocrine therapy	0.48	0.866	0.48	0.846	0.54	0.823	-0.11	0.959	7.37	0.006*
Time	− 0.76	0.597	4.04	0.026*	2.85	0.030 *	-13.16	< 0.001*	10.78	< 0.001*
Pseudo R^2^	**0.05**		**0.10**		**0.11**		**0.19**		**0.20**	

The significance of bold values is to distinguish it from the other entries

*Statistically significant

## Discussion

Women with implant reconstruction were significantly younger than women with total mastectomy (44.9 years versus 52.3 years; *p* < 0.001) or DIEP/FCI reconstruction (49.5 years; *p* = 0.008) (Table 1). Consistent with the literature, the influence of age on HRQoL did not appear to be relevant in the regression models for HRQoL (Tables 4 and 5) [9, 10]. Santosa et al. showed that HRQoL may increase with age after reconstructive procedures [11]. Thus, reconstruction may also be an appropriate option for older patients, especially as the probability of breast reconstruction after mastectomy seems to be lower in older women than in younger patients [12]. One reason may be the surgeon’s disproven assumption that reconstructive surgery in older patient populations is associated with an increased complication rate or an unsatisfactory treatment outcome [13]. BMI was not significantly different in the groups (Table 1) and, in line with the literature, we found a significant lower HRQoL with increasing BMI in the regression models (Tables 4 and 5) [14]. A significantly lower proportion smoked in the DIEP/FCI group compared to the others (*p* = 0.042) (Table 1), as only patients who had stopped smoking at least 6 weeks prior to surgery were considered eligible for this surgical procedure. Overall, 15% (33/227) of patients had a breast cancer-associated gene mutation with 88% (29/33) of them already being confronted with the diagnosis of breast cancer. High-risk genetic status differed significantly between groups (mastectomy (1.4%) < DIEP/FCI (15.8%) < implant (25.6%) (*p* < 0.001)), as women with a genetic high-risk situation are more likely to be diagnosed at a younger age and therefore opt for breast reconstruction. This distribution may partly reflect our institutional policy, which favors primary implant-based reconstruction for carriers of germline pathogenic variants. The histopathologic workup prior to DIEP/FCI reconstruction is considered an oncologically safe approach as occult carcinomas have been reported in up to 11.3% [15].

The frequency of all additional therapies that were applied or completed prior to surgery—except for endocrine therapy—differed significantly between the groups as most patients in the DIEP/FCI group had already completed their acute oncologic therapy. Radiotherapy had a significant negative impact on HRQoL in the FACT-B and in the domain Physical Well-Being (Chest) (*p* < 0.001) at T1 as this therapy leads to local changes, whereas chemotherapy had a significant negative impact on Sexual Well-Being (*p* = 0.044), which may reflect the systemic effects.

In our cohort, preoperative HRQoL was lowest in the DIEP/FCI group across all BREAST-Q domains potentially reflecting the cumulative burden of previous treatments and diagnostic procedures [16–20]. Previous studies have shown that two-stage reconstruction pathways can be associated with increased short-term distress, anxiety, and depressive symptoms compared with single-stage procedures [21, 22]. The frequency of adjuvant therapies after surgery differed significantly between the groups. The highest rate of adjuvant radiation and chemotherapy was observed for mastectomy, which might be the reason for the significantly lower scores in most BREAST-Q domains at T2 in the mastectomy group (Fig. 2). All means of BREAST-Q subsores were within mean ± SD reported by Mundy et al. [23] at T1 and at T2, with the exception of the Physical Well-Being (Chest) domain (Fig. 2).

### Complications

Overall, 11.0% (25/227) of patients had complications within 5 months after surgery that implied an inpatient stay or reoperation. Rising complication rates with increasingly complex and extensive operations (mastectomy 4.1% < implant 11.7% < DIEP/FCI 17.1%) are consistent with the international literature [24, 25]. The rates of total reconstruction loss (1.3% in the implant group and 5.3% in the DIEP/FCI group) also fall within the reported ranges [26, 27].

### Discussion of HRQoL

The DIEP/FCI group recorded a significant increase in HRQoL in the domain Psychosocial Well-Being over 5 months. Since breast reconstruction is often requested by patients in terms of improving Psychosocial Well-Being, this domain was even recommended by Dean et al. as an indicator of HRQoL after breast reconstruction [17]. Although the implant group had no significant increase in the domain Psychosocial Well-Being, these patients were the only ones with a significant increase in the FACT-B domain Emotional Well-Being (*p* = 0.032) and also showed the highest preoperative BREAST-Q scores in all but one domain (Physical Well-Being (Chest)). A postoperative decrease in HRQoL in Physical Well-Being (Chest) has already been mentioned in other studies but should be closely monitored in the prospective course of the study [28, 29].

Looking at the domain Functional Well-Being, Nissan et al. showed that it was even higher after mastectomy than after reconstruction and that the negative effects of reconstruction lasted up to 18 months [30]. In the present study, there also was a non-significant increase in Functional Well-Being in the FACT-B in the mastectomy group (Fig. 3). The DIEP/FCI group recorded the highest scores in the domain Functional Well-Being pre- and postoperatively. As expected [31], scores decreased significantly in the domain Physical Well-Being (Abdomen) after 5 months in the DIEP/FCI group and should be monitored as the study progresses. Time was found to be a significant factor for an increase in HRQoL in the BREAST-Q domains Satisfaction with Breast and Psychosocial Well-Being, but it had a significant negative impact on Physical Well-Being (Chest).

### Limitations of the study

The major limitation of the REKO 001-trial is the lack of randomization and the comparison of patients receiving either immediate or delayed reconstruction. In the DIEP/FCI group, approximately 90% of patients had previously undergone mastectomy or implant reconstruction, contributing to the lower baseline HRQoL at study entry. Although prior breast surgery was included in the regression models and showed no significant association with HRQoL (except for Sexual Well-Being), the timing of reconstruction remains an important confounder. Acute, prospectively ongoing oncologic treatment likely affects HRQoL differently than the post-therapeutic state of patients presenting for delayed reconstruction. This fundamental difference must be accounted for when interpreting group comparisons.

Another limitation is that surgery for breast cancer is analyzed together with surgery for risk reduction in carriers of germline pathogenic variants. Mutation carriers have a different approach to surgery and different expectations.

When characterizing the patient collective, only the current smoking status but not the smoking history was evaluated, which can have an impact on the outcome of surgery, healing, complications, and HRQoL.

## Conclusion

Patients with DIEP/FCI reconstruction started with the lowest baseline HRQoL (BREAST-Q) preoperatively yet showed an increase within the first 5 months after surgery. Patients with implant-based reconstruction demonstrated higher HRQoL both before and after surgery compared with those undergoing mastectomy. Moreover, the implant group showed a significant increase in Emotional Well-Being (FACT-B), whereas HRQoL declined in most domains after mastectomy. These results should be interpreted with consideration of reconstruction timing as a potential confounder.

The increased complexity of autologous procedures was associated with higher complication rates; therefore, patients considering DIEP/FCI reconstruction should be aware of the increased risk of complications and of the early postoperative decrease in Abdominal Well-Being. Time was identified as a relevant factor influencing HRQoL, making the long-term follow-up at 2, 5, 7, and 10 years essential for understanding how HRQoL evolves in each group.

## Data Availability

No datasets were generated or analysed during the current study.
